# Enhancement of eruption explosivity by heterogeneous bubble nucleation triggered by magma mingling

**DOI:** 10.1038/s41598-017-17098-3

**Published:** 2017-12-04

**Authors:** Joali Paredes-Mariño, Katherine J. Dobson, Gianluigi Ortenzi, Ulrich Kueppers, Daniele Morgavi, Maurizio Petrelli, Kai-Uwe Hess, Kathrin Laeger, Massimiliano Porreca, Adriano Pimentel, Diego Perugini

**Affiliations:** 1 Department of Physics and Geology, University of Perugia, Piazza dell’Universitá, 06100 Perugia, Italy; 2 Department of Earth Sciences, Durham University, Science Labs, Durham, DH1 3LE United Kingdom; 3 Institute of Planetary Research, German Aerospace Center, Planetary Physics, Rutherfordstraße 2, 12489 Berlin, Germany; 4 Department of Earth and Environmental Sciences, Ludwig-Maximilians-Universität, Theresienstraβe 41, 80333 Munich, Germany; 5 Centro de Informação e Vigilância Sismovulcânica dos Açores, Rua Mãe de Deus, 9501-801 Ponta Delgada, Portugal; 6 Instituto de Investigação em Vulcanologia e Avaliação de Riscos, University of the Azores, Rua Mãe de Deus, 9501-801 Ponta Delgada, Portugal

## Abstract

We present new evidence that shows magma mingling can be a key process during highly explosive eruptions. Using fractal analysis of the size distribution of trachybasaltic fragments found on the inner walls of bubbles in trachytic pumices, we show that the more mafic component underwent fracturing during quenching against the trachyte. We propose a new mechanism for how this magmatic interaction at depth triggered rapid heterogeneous bubble nucleation and growth and could have enhanced eruption explosivity. We argue that the data support a further, and hitherto unreported contribution of magma mingling to highly explosive eruptions. This has implications for hazard assessment for those volcanoes in which evidence of magma mingling exists.

## Introduction

The intrusion of mafic magma into a more evolved magma chamber is one of the main processes responsible for triggering highly explosive volcanic eruptions^1–3^. This process increases the volumetric stress in the chamber, and may drive volatile transfer from the mafic to the felsic magma which, when coupled to the additional thermal input from the mafic magma, destabilises the magmatic system and triggers rapid volatile exsolution and eruption^1–7^.

Fractal geometry methods have been widely applied in geosciences, and among the applications has been the study of fragmentation of Earth materials, where the fractal dimension (*D*) represents a powerful tool to characterize the fragmentation process [e.g. fault gauge development^8,9^, subsidence breccias^10^, and rock fragmentation^11–13^. In volcanology, fractal statistics are applied, for example, to study ash morphology and fragment size distributions to discriminate magma fragmentation and pyroclastic transport processes and to derive empirical relationships linking the energy available for fragmentation and the fractal exponent^14–22^. Recently, fractal analysis of the size distributions of mafic enclaves dispersed in felsic magmas has shed new light upon the mechanisms operating during magma chamber refilling associated with initiating eruptions^23–25^. These studies reveal mafic fragments archive important information about the magma interaction processes and its role as eruption trigger, which is impossible to investigate by direct observation.

Here we present new data that allow understanding of the processes that led to the dispersion of mafic fragments throughout a more felsic magma, and apply fractal statistics to understand the processes leading to fragmentation of the mafic magma and its role enhancing and facilitating volcanic explosions.

## Results

**Figure 1:**
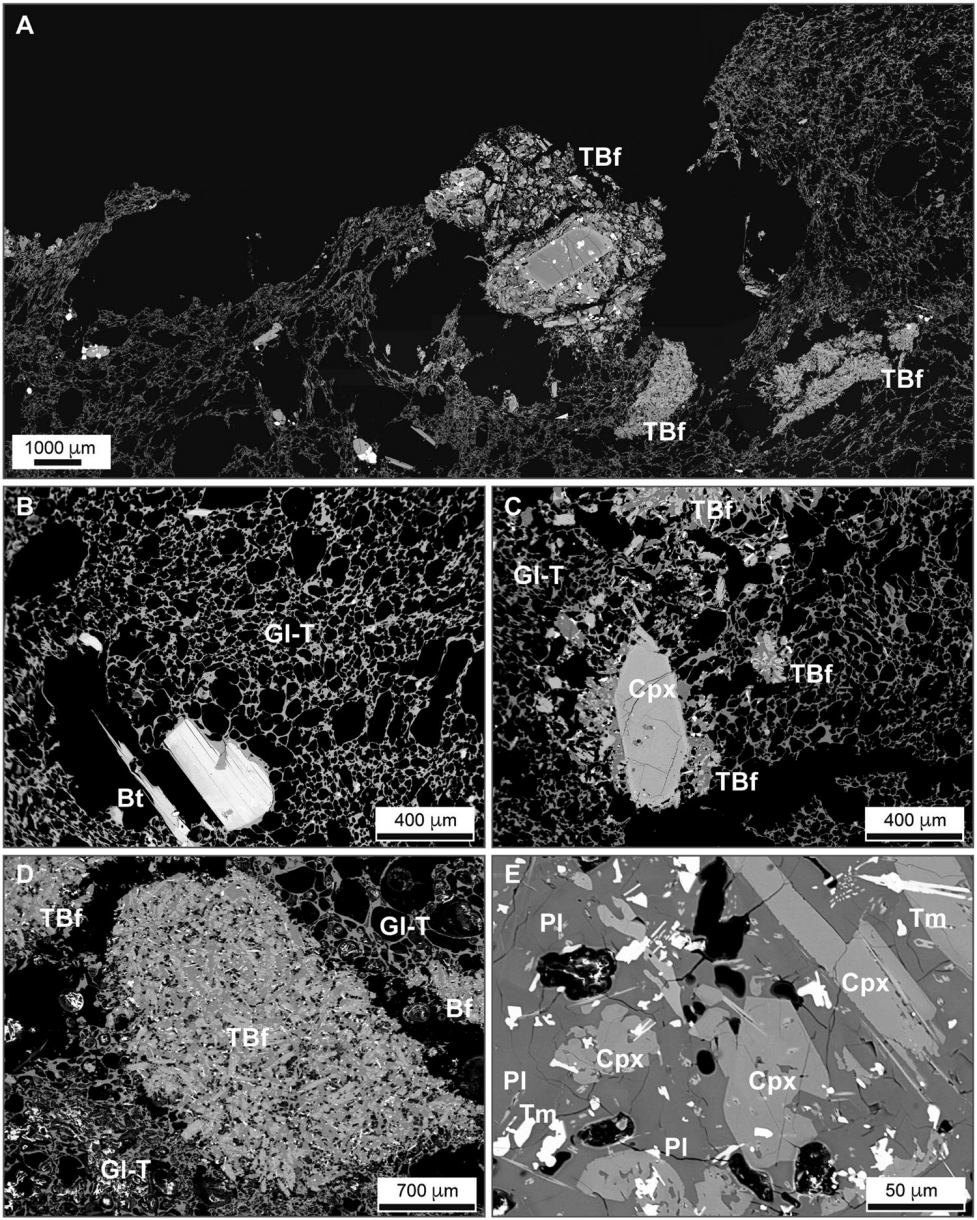
Representative back-scattered electron images showing the main petrographic features of studied rocks. (**A**) General view of a studied sample showing the occurrence of trachybasaltic fragments in the trachytic pumice; (**B**) trachytic pumice with glassy vesicular groundmass (Gl-T) and rare crystals of biotite (Bt); (**C**) trachybasaltic fragments (TBf) with clinopyroxene crystal (Cpx), immersed in the glassy (Gl-T) trachytic pumice; (**D**,**E**) zoomed-in views of trachybasaltic fragments showing undercooling textures. A few vesicles (dark rounded areas) are also present in the trachybasaltic fragment. Tm: titanomagnetite; Pl: plagioclase.

Analysed samples come from the Upper Member of the Santa Bárbara Formation (see Supplementary information), on the NE flank of Sete Cidades volcano, São Miguel, Azores, a pumice fall deposit from the last paroxysmal event related to the caldera formation at Sete Cidades, 16 ky BP^26,27^. This formation contains white to yellow trachytic pumice clasts that contain fragments of trachybasaltic composition. These textures are regarded as the product of magma interaction between a trachytic and a trachybasaltic magma^27,28^. The pumice is highly vesicular (>75.0 vol% vesicularity) and mostly aphyric, with a few crystals of alkali-feldspar (ca. 1.5 vol%) and biotite (ca. 0.5 vol%) (Fig. 1). The trachybasaltic fragments themselves show cuspate margins and sharp contact with the surrounding trachytic glass (Fig. 1). They have lower vesicularity (<10.0 vol% on average) and are fine-grained, with a diktytaxitic groundmass of feldspar (alkali-feldspar and plagioclase), kaersutite, clinopyroxene, Fe-Ti-oxides (ilmenite and magnetite) in a decreasing order of abundance and interstitial glass. Skeletal and/or acicular crystal morphologies and swallowtail plagioclases are common (Fig. 1).

**Figure 2:**
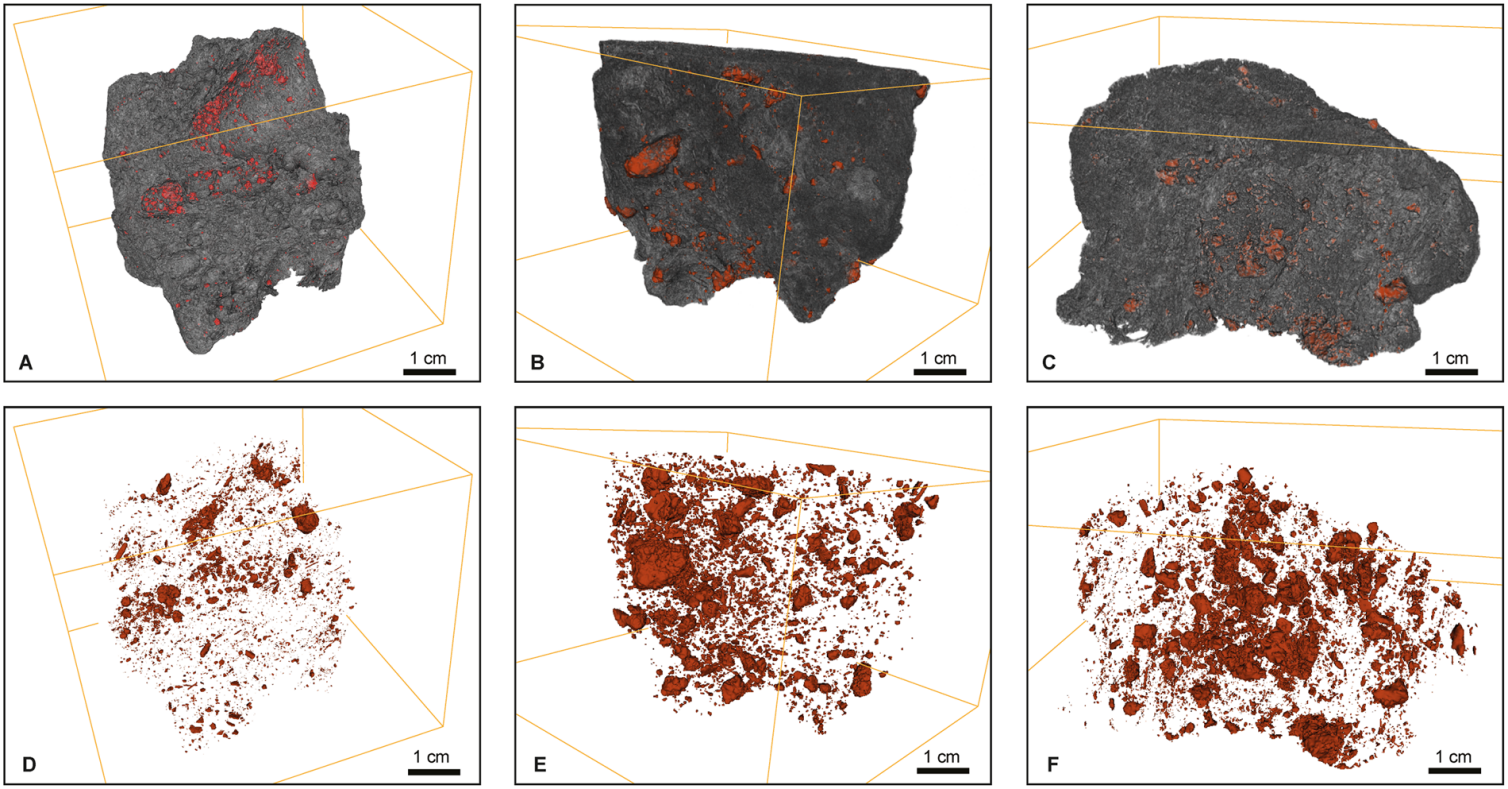
(**A**–**C**) 3D reconstruction of studied samples (A: sample 073UK; B: sample 074UK; C: sample 075UK; see Supplementary information). Trachytic pumice and trachybasaltic fragments are reported in the grey and red colour, respectively; (**D**–**F**) 3D distribution of trachybasaltic fragments (reported in the red colour) from the same pictures reported in the upper panels, after removal of the trachytic component.

**Figure 3:**
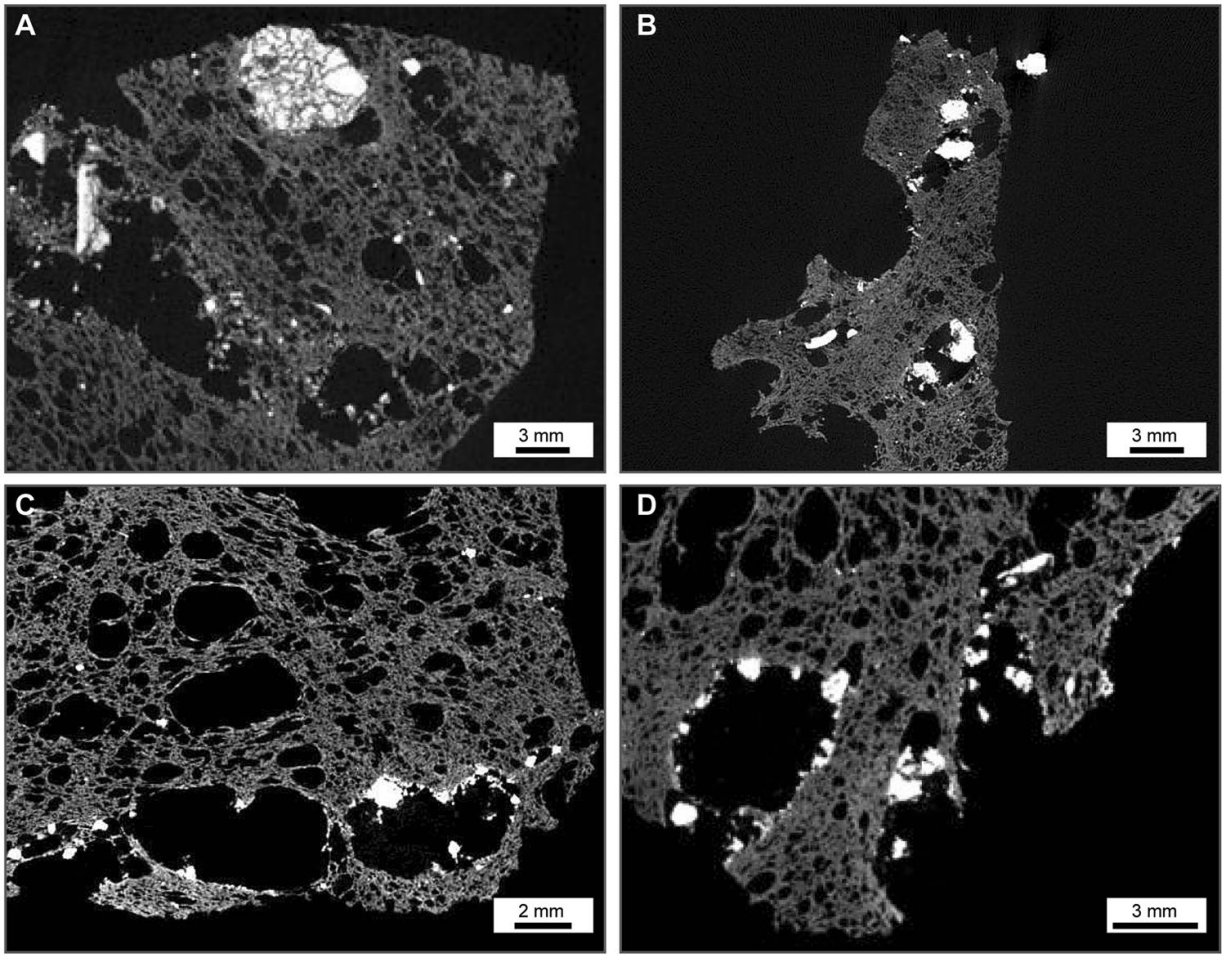
Representative slices of studied samples extracted from the reconstructed 3D volumes. (**A**) Intensely fractured trachybasaltic fragment whose fragments were not pulled apart by the growing bubbles in the trachytic melt; (**B**–**D**) Distribution of trachybasaltic fragments coating the inner walls of bubbles in the trachytic pumices.

**Figure 4:**
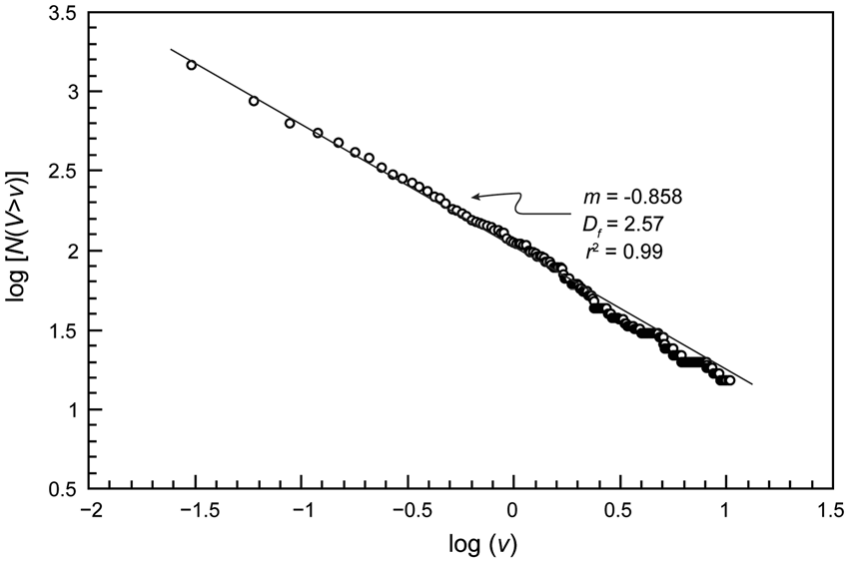
Variation of the logarithm of cumulative number of trachybasaltic fragments with volumes *V* larger than comparative volume *v* (log[*N*(*V* > *v*)]) against log(*v*) according to Eq. [3]. In the graph, the value of *r* ^2^ from the linear fitting, and values of *m* and *D*_*f*_ are also reported.

The 2D (Fig. 1) and 3D (Fig. 2) images show a variety of complex textures. Animations showing the 3D rendered data are provided as Supplementary information. The images show that the majority of trachybasaltic fragments are commonly distributed across the inner surfaces of bubbles (Fig. 3). The average amount of total bubbles containing trachybasaltic fragments is on the order of 40.0 vol%. Larger fragments are intensely fractured (Fig. 3A) with the size distribution of the jig-saw fragments being comparable to that of the fragments found dispersed across the internal surfaces of the bubbles (Fig. 3B–D). 3D renderings of pumice clasts show a widespread and even distribution of the fragments within the pumice, and visual inspection suggests that almost all larger bubbles (i.e. bubbles with average diameter larger than 3.0–4.0 mm) are associated with trachybasaltic fragments (Figs 2 and 3D animations in the Supplementary information). As the analysed clasts belong to the same pumice fall deposit, the volume distribution data for all three clasts are combined prior to the fractal analysis (Fig. 4, plotted after Eq. [3], see Methods section), yielding a straight line and fulfilling the requirement for a fractal-fragmented distribution^29^. Linear interpolation of data gives a slope (*m*) of −0.858, and a fractal dimension *D*_*f*_ = 2.57 (Eq. [4]; Methods section).

## Discussion

The conceptual model of a fractal-fragmented population is the self-similar fragmentation of a mass into progressively smaller particles^11,12,29^. In this model the direct contact between two fragments of near equal size will result in the breakup of one block^29,30^ (see Supplementary information). A particle distribution will therefore evolve towards a minimum number of particles at any size. The model yields a fractal dimension of *D*_*f*_ = 2.60 (see Supplementary information), and from observations of a number of rock types, this appears to be a typical value for fragmentation of materials in the brittle regime^29^. The measured fractal dimension of fragmentation for the trachybasaltic particles (*D*_*f*_ = 2.57) indicates a single fragmentation mechanism, and the good agreement with this model value implies that the trachybasalt also underwent solid-state (brittle) fragmentation. This is corroborated by the generally sharp edges and cuspate margins of the trachybasaltic fragments (Figs 1–3).

### A model for magma interaction and fragmentation

Here we endeavor to develop a model for the mingling process and the subsequent fragmentation of the trachybasalt within the trachytic magma^27,28^. Texturally, this can be observed by the presence of trachybasaltic fragments attached to the inner wall of large bubbles in the pumice (Figs 1–3). As a consequence, the contact between the two magmas must have occurred at depth, prior to nucleation and growth of the bubbles in the trachyte. In the following, we are discussing different hypotheses that might explain these features.

A first hypothesis might be that the trachybasalt was already present at depth as a solidified magma body and was crosscut by the ascending trachytic magma. At this stage, trachybasaltic fragments were incorporated into the trachytic melt and transported towards the surface. When the molten trachyte, and its solid cargo, represented by the fragments of trachybasalt, reached the level at which bubbles started to nucleate, the trachybasalt was captured by the growing bubbles and remained “glued” in their inner walls. Some considerations rule out this hypothesis as the process responsible for the observed structures. In fact, the trachybasaltic fragments show clear signs of strong undercooling (Fig. 1D,E)^31–36^, arguing against a solidification of the trachybasaltic magma at depth. Indeed, such a process would have generated textures tending to those typically of plutonic or sub-volcanic rocks rather than the undercooling textures observed in the trachybasaltic fragments.

A second hypothesis could be the explosion of trachybasaltic melt blobs against the host magma at the fragmentation level, upon decompression. This hypothesis requires that the trachybasalt was dispersed into the trachyte in a magmatic state and was able to deform and eventually explode against the walls of the bubbles when the ascending magmatic mixture reached the fragmentation level. A possible scenario is that the trachybasaltic magma was injected into the trachytic magma at depth^3,37–41^. During this process, heat was transferred to the trachytic magma and its viscosity was reduced. Entrainment of mafic magma might have been favored by buoyant rise of vesiculated mafic blobs that would become dispersed into the trachytic magma^42^. However, there are two main facts opposing to this idea: (1) the explosion of the trachybasaltic droplets requires that they must have vesiculated vigorously leading to their fragmentation. Textural analysis of the trachybasaltic fragments indicates a very low vesicularity (<10.0 vol%) and no evidence of break-up of bubbles at the clast boundaries; (2) the strong undercooling of the trachybasalt after its contact with the trachytic magma (as testified for example by the acicular/skeletal crystals and the interstitial glass in the mafic fragments) likely limited the deformability of the trachybasaltic blobs, rapidly bringing them towards a solid state. Accordingly, it seems unlikely that the dispersion of trachybasaltic fragments at the inner walls of bubbles in the trachyte is due to the explosion of the trachybasalt.

A further hypothesis might be that the trachybasaltic magma was injected into the trachytic magma body at depth, where it quenched rapidly^33,43,44^ and underwent brittle fragmentation. This hypothesis is supported by the common occurrence in felsic rocks of mafic enclaves with highly variable fragment size distributions^23–25^, as in the case of the studied Sete Cidades rocks. Additional indications for the appropriateness of this hypothesis can be found in the petrographic features of the trachybasaltic fragments. Disequilibrium textures of mineral phases indicate that the trachybasaltic magma underwent strong undercooling^33,36^, a feature corroborated by the presence of a fine-grained groundmass and the interstitial glass in the trachybasaltic fragments. These features agree well with observations of rocks in which mafic magmas were quenched during their injection into more felsic host melts^33,43,44^. The main process for the formation of the observed undercooling textures is, therefore, to be attributed to the temperature difference between the trachybasaltic and the trachytic magma. The rapid quenching moved the rheological behaviour of the mafic component towards that of a solid. Support to this interpretation is provided by the results from fractal analysis where the value of fractal dimension of fragmentation (*D*_*f*_ = 2.57) indicates that the trachybasaltic component was in the solid state soon after it was dispersed into the trachytic melt. The fact that this value of fractal dimension is very similar to *D*_*f*_ values (*D*_*f*_ = 2.50–2.55) estimated for size distributions of mafic enclaves dispersed in felsic magmas in both the volcanic and plutonic environment^23^, corroborates the idea that the above envisaged processes adequately explains the features observed in the studied rocks.

**Figure 5:**
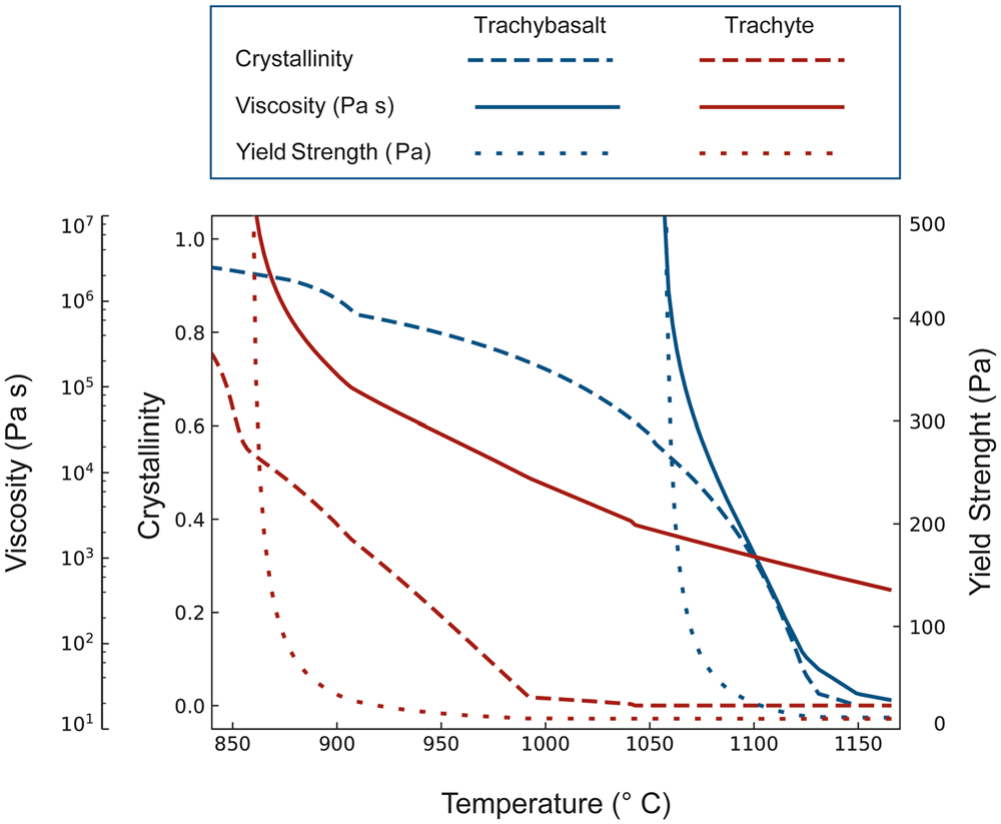
Variation of crystallinity, magma viscosity and yield strength as a function of temperature for the trachytic and trachybasaltic magmas (see Methods section).

**Figure 6:**
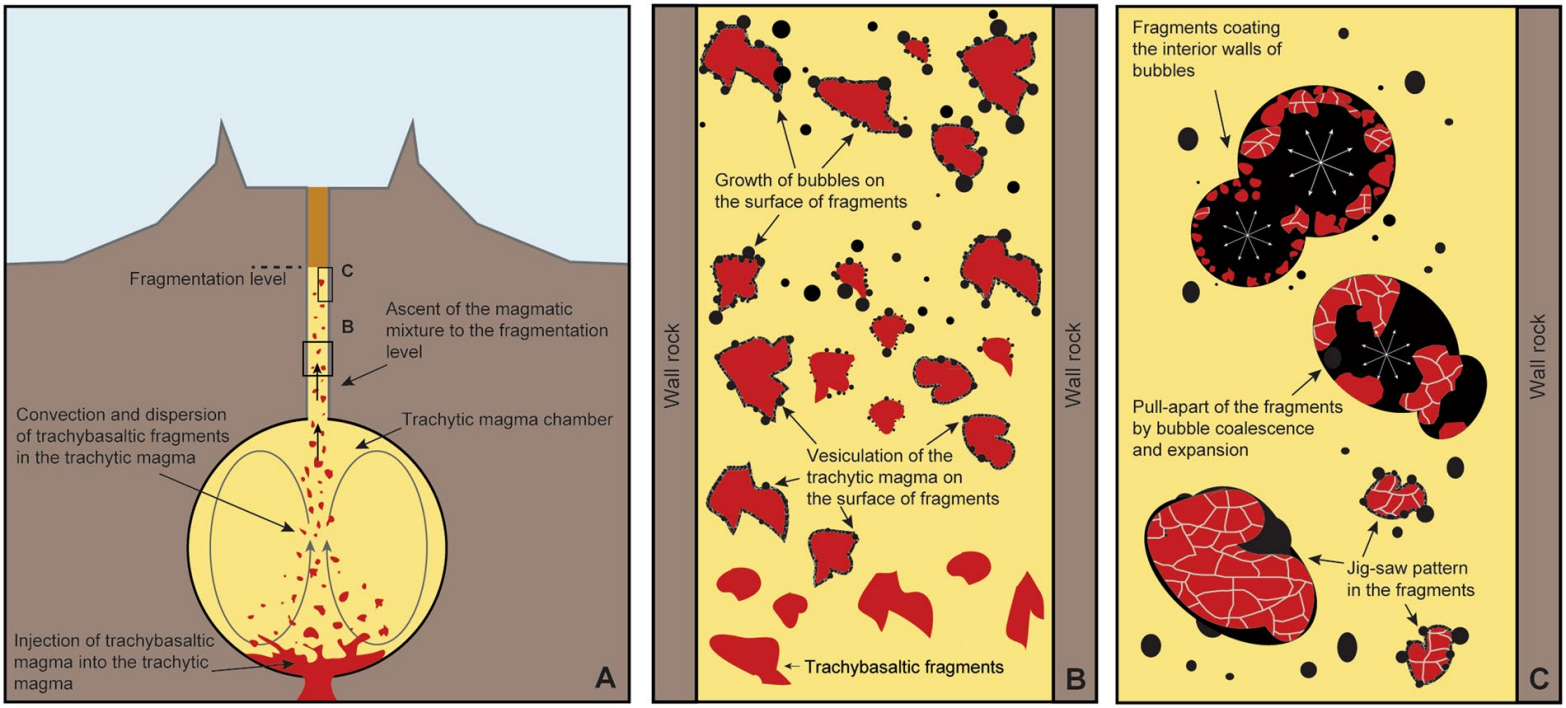
Synoptic scheme of the evolution of the magmatic system from the injection of the trachybasaltic magma into the trachytic chamber to the fragmentation level in the volcanic conduit. (**A**) The injection of the trachybasaltic magma in the trachytic chamber generated thermodynamical instability. The trachybasaltic magma underwent strong undercooling and fragmentation. At the same time the heat provided by the trachybasalt triggered convection dynamics facilitating the mobility of the magmatic system that migrated towards shallower levels; (**B**) zoomed-in view of the system during the magma migration in the conduit: trachybasaltic fragments acted as favourable sites for bubble nucleation in the trachytic melt; (**C**) growth of bubbles around the trachybasaltic fragments provoked the detachment of smaller pieces of trachybasaltic rock that remained attached to the inner walls of the bubbles that formed in the trachytic melt.

Rheological and thermal models can aid in tracking quantitatively the evolution of the studied system during magma interaction^45,46^ (see Methods section). Figure 5 reports the variation of the rheological behaviour (viscosity and yield strength) and crystallinity of the trachytic and the trachybasaltic magma as a function of temperature (see Methods section). We consider a trachytic magma mass with a crystallinity of ca. 2.0 vol% (as inferred from petrography), located at a depth of ca. 3.5–4.0 km and with a water content of 2.0 wt%^28^. At these conditions, the temperature of the trachyte is ca. 990 °C corresponding to a viscosity of ca. 10^4^ Pa s (Fig. 5). We assume this temperature as the temperature of the trachytic host magma when the injection of the trachybasalt occurred (Table 1). As inferred from petrographic observations, the trachybasalt has a glassy/microcrystalline groundmass constituted by undercooled textures, which formed when it came into contact with the trachytic magma. Therefore, we consider the injection of the trachybasalt at a temperature close to its liquidus temperature (i.e. ca. 1160 °C; Fig. 5; Table 1). As the trachybasalt is injected into the trachyte, it undergoes cooling and crystallizes. As temperature of the trachybasalt decreases, its viscosity and yield strength increase (Fig. 5). At temperature of ca. 1060 °C the trachybasalt is effectively solid (viscosity >10^7^ Pa s and yield strength >500 Pa; see Methods section) whereas the trachyte can still fluidly deform (Fig. 5). Therefore, we consider this temperature of the trachybasaltic magma as the temperature at which it started fragmenting. These results can be used to estimate the volume proportions of the two magmas that interacted before the eruption. In particular, using the approach provided by Folch and Martì^47^, the volume ratio of two magmas can be estimated by knowing the decrease in temperature of the trachybasaltic magma (Δ*T*_*m*_; Eq. 5; Methods section). As reported above, in our case Δ*T*_*m*_ is equal to 100 °C, leading to a volume ratio of the two magmas φ=0.55 (see Methods section). This φ value corresponds to approximately 35 vol% of trachybasaltic magma and 65 vol% of trachytic magma. These can be considered as the volume proportions of the two magmas that interacted at depth and that mobilized the magmatic system forcing its ascent towards the Earth surface and triggering the eruption (Fig. 6A).

**Table 1:** Whole rock chemical composition and physical properties of the end-members (trachybasalt and trachyte) used in the rheological and thermal modelling (see Methods section).

	Unit	Trachybasalt	Trachyte
SiO_2_	wt.%	48.12	62.39
TiO_2_	wt.%	3.69	0.91
Al_2_O_3_	wt.%	15.90	17.87
FeO_t_	wt.%	10.91	3.97
MnO	wt.%	0.21	0.20
MgO	wt.%	4.71	0.86
CaO	wt.%	9.25	1.59
Na_2_O	wt.%	3.93	6.63
K_2_O	wt.%	2.00	5.35
P_2_O_5_	wt.%	1.29	0.22
Tot	wt.%	100.00	100.00
H_2_O	wt.%	0.50	2.00
Cp	J k^−1^ kg^−1^	1200	1000
ρ	Kg/m^3^	2650	2340
T_liquidus_	°C	1160	1040

*Cp*, specific heat^47^; *ρ*, density; *T*_liquidus_, liquidus temperature. *ρ* and *T*_liquidus_ values were calculated using the software MELTS^62,63^.

During magma ascent following mingling, heterogeneous nucleation of bubbles occurred preferentially on the defects provided by the trachybasaltic fragments (Fig. 6B), driving rapid and vigorous eruption. This idea is corroborated by both experimental and field studies suggesting heterogeneous nucleation of bubbles on magnetite, silicate phases and xenoliths^48–58^. In our case, although heterogeneous nucleation might have also occurred on crystals in the trachytic magma, their low amount (of the order of 2.0 vol%) compared to the amount of trachybasaltic fragments (of the order of 15.0–20 vol%), suggests that this process must have played a very minor role. This also indicates that vesiculation and bubble growth can be strongly enhanced by the presence of solid fragments in almost aphyric magmas, as in the trachyte studied here.

Bubble expansion and coalescence during decompression, and explosion as the ascending magma reached the fragmentation level, then drove the separation of the already highly fragmented trachybasalt and final dispersal of the trachybasaltic fragments, and the fragmentation of the pumice (Fig. 6C).

Therefore, from our study it can be hypothesised that trachybasaltic fragments might have acted as energetically favourable sites to trigger bubble nucleation and growth in the trachytic melt. This would have facilitated gas exsolution from the trachyte, consequently enhancing the explosivity of the trachyte. In particular, gas exsolution from the trachyte might have occurred at lower degree of oversaturation, corresponding to greater depth. This way, the volume increase may have triggered an accelerated ascent of the magma. The vesiculation on the trachybasaltic fragments was, therefore, a point of no return and an eruption became unavoidable. This indicates a further possible contribution of magma mingling in triggering explosive eruptions, which remained unnoticed up to now. We believe these results will shed new light on the complex interplay of processes operating in determining bubble formation during magma ascent and eruption. In particular, volcanic eruptions characterized by large lithic contents (i.e. mafic enclaves, xenoliths, etc.) might develop more vigorously relative to those eruptions in which the lithic content is lower. In the light of data reported here, detailed field work on pyroclastic deposits needs to be carried out in order to assess the importance of this process, for example, on eruption style and ash dispersal. In particular, the lithic/juvenile content ratios could be measured in proximal deposits and compared to the grain size distribution of juvenile material in the distal deposits, in order to derive relations between the lithic content and the ash dispersal ability of the eruption. This, combined with new decompression experiments of silicate melts with variable lithic contents, designed to quantify the role of solid materials in the magma on the efficiency of bubble nucleation and growth, might represent a decisive step to better understand how explosivity can be modulated by magma mingling in volcanic eruptions.

## Methods

### X-ray micro tomography (XMT) acquisition and processing

The XMT analysis was performed on a GE v|tome|x s^©^ microfocal system, operating at a maximum accelerating voltage of 80 kV (250 µA) and using a 0.1 mm Cu filter to minimise beam hardening. The 3D volumes were reconstructed using GE proprietary software from 1000 projections. Each projection was acquired using two seconds exposure, two frames averaging and detector shift for noise and ring reduction, respectively. A nominal voxel size of 50 µm was achieved for all three samples. Uncertainty in volume determinations on any individual feature is estimated to be 5% for features with volume greater than about 100 voxels (equivalent to 500 µm × 500 µm × 500 µm) because of the reduced precision of the phase edges^59^. Visualization and quantification was performed using the Avizo^©^ software.

The thinnest films that make up some bubble walls are beyond image resolution, as will be the smaller bubbles in the population, and the detail of the diktytaxitic texture in the trachybasaltic fragments. No noise reduction filtering was used, and all three clasts were processed using the same algorithms and parameters. Trachybasaltic fragments were segmented from the host trachyte using an iterative procedure applying an automatic moment-preserving bi-level thresholding^60^ to sequentially separate the brightest phase from the trachytic pumice based on the peaks in the greyscale histogram. A total of ca. 3.0 × 10^4^ fragments were analysed and results show a large variability of size (volume) ranging from ca. 1.0 to ca. 2 × 10^–3^ mm^3^.

### Fractal analysis

Fractal analysis is applied to study the fragment size distribution of trachybasaltic fragments, on the XMT derived 3D volume data, as explained below.

In the light of fractal theory, Mandelbrot^61^ has shown that fractal fragmentation could be quantified by measuring the fractal dimension of fragment population through the equation:

1 $$N(R > r)=k{r}^{-{D}_{f}}$$

where *D*_*f*_ is the fragmentation fractal dimension; *N*(*R* > *r*) is the total number of particles with linear dimension *R* greater than a given comparative size *r*, and *k* is a proportionality constant. Taking the logarithm of both sides of Eq. [1] yields a linear relationship between *N*(*R* > *r*) and *r* with *D*_*f*_ related to the slope coefficient, *m*, by

2 $${D}_{f}=-m$$

Eq. [1] is based on linear size comparisons, i.e. *R* > *r*. If the basis for size comparison is taken as ‘volume’ (*V* > *v*), as in the case of the studied trachybasaltic fragments, Eq. [1] becomes

3 $$N(V > v)=k{v}^{-{D}_{f}/3}$$

with

4 $${D}_{f}=-3m$$

since the linear extent of volume is the cubic-root of area *(A* ^*l/3*^ *)*.

Fractal dimension (*D*_*f*_) derived from Eq. [1] is a measure of the size-number relationship of the particle population or, in other terms, the fragmentation of the population.

### Rheological and thermal modelling

The rheological evolution of the magmatic system was modelled starting from the whole rock compositions of the two end-members reported in Table 1, representing the most and least evolved compositions measured on the studied samples. Crystallization paths were calculated using MELTS^62,63^ considering a depth of the magma chamber, where the mixing process started, located at approximately 3.5–4.0 km in accordance with Beier^28^. In the calculations, a water content of 2.0 wt% and 0.5 wt% was used for the trachyte and trachybasalt, respectively^28^. Liquidus temperature of the trachytic and trachybasaltic magmas, calculated using MELTS, are 1040 °C and 1160 °C, respectively (Table 1). Viscosities of the melt and melt plus crystals were calculated using the method of Giordano *et al*.^64^ and Mader *et al*.^65^, respectively. Yield strength of magmas was estimated following the approach reported in Pinkerton and Stevenson^66^.

The volumes of magmas were estimated using the approach reported in Folch and Martì^47^ using the following equation

5 $${\rm{\Delta }}{T}_{m}=\frac{{\rho }_{f}{C}_{f}}{\phi {\rho }_{m}{C}_{m}+{\rho }_{f}{C}_{f}}\,({T}_{fi}-{T}_{mi})$$

where *T*_*mi*_ and *T*_*fi*_ are, respectively, the initial temperatures of the trachybasaltic and trachytic magmas, *C*_*m*_ and *C*_*f*_ their specific heat capacities, *ρ*_*m*_ and *ρ*_*f*_ their densities, and *φ* = *V*_*mi*_/*V*_*fi*_ (where *V*_*mi*_ is the volume of injected mafic magma and *V*_*fi*_ is the volume of the felsic magma in the chamber)^47^. Parameters used in the calculations are given in Table 1. By knowing the value of Δ*T*_*m*_, resulting from the cooling of the trachybasaltic magma from liquidus temperature to the temperature at which it reaches a solid state behaviour (i.e. Δ*T*_*m*_ = 100 °C, corresponding to a crystallinity of ca. 55%, viscosity >10^7^ Pa s and yield strength >500 Pa; Fig. 5), the volume ratio between magmas (*φ*) and their relative proportions were estimated.

## Electronic supplementary material

Supplementary information

Video - Sample_073_UK - SI - Movie 1

Video - Sample_073_UK - SI - Movie 2

Video - Sample_073_UK - SI - Movie 3

Video - Sample_074_UK - SI - Movie 1

Video - Sample_074_UK - SI - Movie 2

Video - Sample_074_UK - SI - Movie 3

Video - Sample_075_UK - SI - Movie 3

Video - Sample_075_UK - SI - Movie 2

Video - Sample_075_UK - SI - Movie 1
