# Supplementary material for: Enhancement of eruption explosivity by heterogeneous bubble nucleation triggered by magma mingling
Source: Sci Rep. 2017 Dec 4;7:16897. doi: 10.1038/s41598-017-17098-3 (PMC5714953; doi:10.1038/s41598-017-17098-3)
Supplement: Supplementary file 1 — Supplementary information [file 41598_2017_17098_MOESM1_ESM.pdf]

Supplementary information

Enhancement of eruption explosivity by heterogeneous bubble nucleation triggered by magma mingling

Joali Paredes-Mariño[1], Katherine J. Dobson[2], Gianluigi Ortenzi[3], Ulrich Kueppers[4], Daniele Morgavi[1], Maurizio Petrelli[1], Kai-Uwe Hess[4], Kathrin Laeger[1], Massimiliano Porreca[1], Adriano Pimentel[5,6] & Diego Perugini[*1]

[1] *Department of Physics and Geology, University of Perugia, Piazza dell'Universitá, 06100 Perugia, Italy.*

[2] *Department of Earth Sciences, Durham University, Science Labs, Durham DH1 3LE, United Kingdom*

[3] *Institute of Planetary Research, German Aerospace Center, Planetary Physics, Rutherfordstraße 2, 12489 Berlin, Germany.*

[4] *Department of Earth and Environmental Sciences, Ludwig-Maximilians-Universität, Theresienstraße 41, 80333 Munich, Germany*

[5] *Centro de Informação e Vigilância Sismovulcânica dos Açores, Rua Mãe de Deus, 9501-801 Ponta Delgada, Portugal*

[6] *Instituto de Investigação em Vulcanologia e Avaliação de Riscos, University of the Azores, Rua Mãe de Deus, 9501-801 Ponta Delgada, Portugal*

Geological background and sample description

The samples come from the Upper Member of the Santa Bárbara Formation, on the NE flank of Sete Cidades, São Miguel, Azores (Fig. S1), a pumice fall deposit from the last paroxysmal event related to the caldera formation at Sete Cidades, 16 ky BP[1-3].

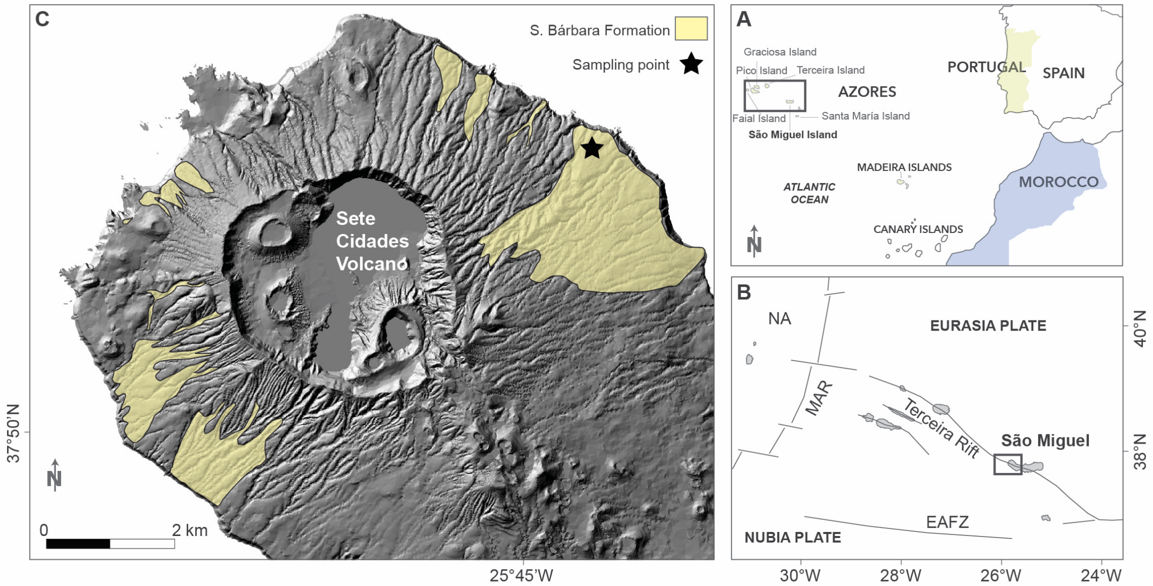

Figure S1: Map of sampling location. A) Regional map for the location of the Azores archipelago at the North Atlantic Ocean; B) Location of São Miguel in the Azores archipelago with indication of the major tectonic structures: MAR - Mid-Atlantic Ridge, NA - North-America plate, EAFZ - East Azores Fracture Zone; C) Digital elevation model of the western sector of São Miguel Island (Azores) showing the areal distribution of Santa Bárbara Formation deposits. Studied samples were collected from the site indicated by the black star. The map layout was built using the open source QGIS (version 2.14.3-Essen; http://www.qgis.org/).

Sete Cidades evolved from a pre-caldera alkali basaltic effusive shield-building phase that gradually evolved to more differentiated compositions (>210 ky), before caldera-forming Plinian to sub-Plinan eruptions of trachytic composition (36, 29 and 16 ky BP) and a post-caldera phase of trachytic magma and minor basaltic flank eruptions[1-8].

The stratigraphy highlights repeated cycling between basaltic to trachytic compositions[3,5,6]. The Upper Member of the Santa Bárbara Formation typically contains white to yellow trachytic pumice clasts that contain fragments of trachybasaltic composition[1].

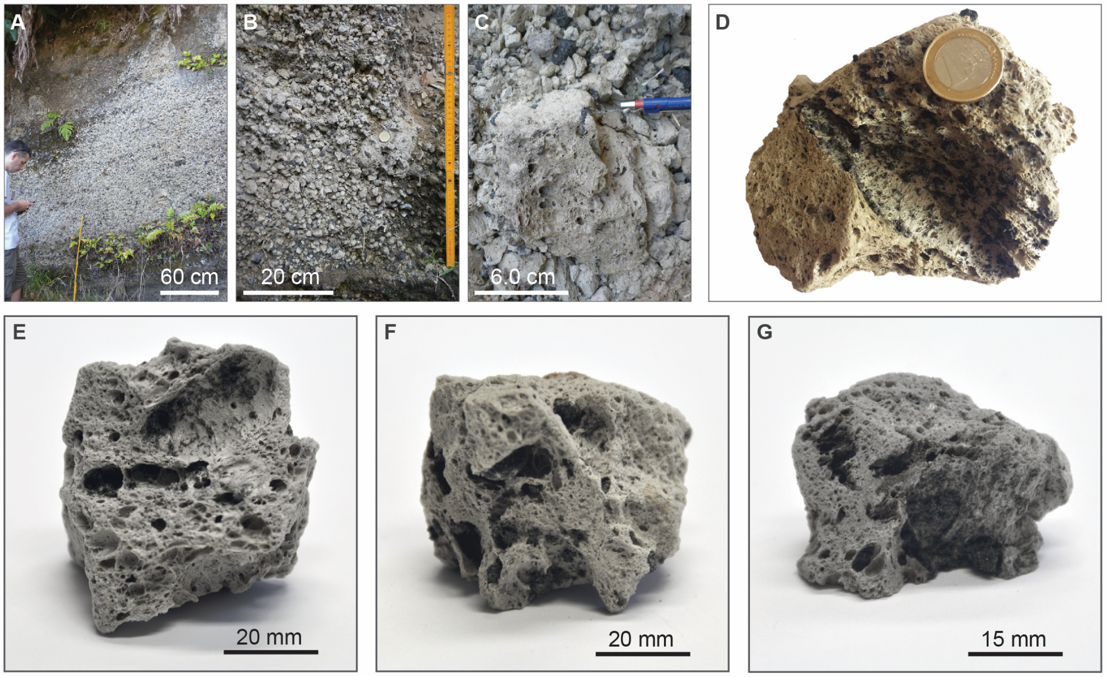

Figure S2: Pictures taken at increasing magnifications (from A to C) showing the pumice fall deposit of the Upper Member of Santa Bárbara Formation from which studied samples were collected. A) General aspect of the fall deposit; B-C) zoomed-in view showing the occurrence of trachytic pumices in which trachybasaltic fragments were dispersed; D) large bubble at the surface of a pumice clast coated by the trachybasaltic fragments; E-G) pictures of the three pumice clasts studied with the 3D XMT.

Conceptual model for fractal fragmentation

The conceptual model used to derive Eq. [3] (see Methods section) is based on the self-similar fragmentation of a mass into progressively smaller particles[9,10]. A possible

fragmentation model is illustrated in Fig. S3A, where two diagonally opposed blocks are retained at each scale[11]. This corresponds to the comminution model proposed by Sammis et al.[12] for fragmentation of solid materials.

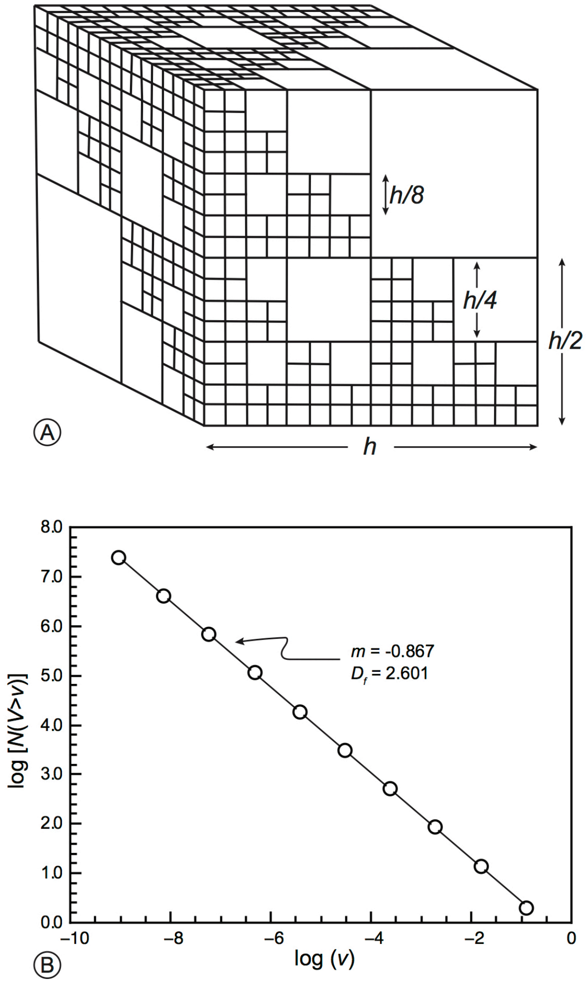

Figure S3: A) Schematic representation of fractal fragmentation. A zero order cubic cell with dimension *h* is divided into eight cubic elements with dimension *h*/2. The fragments with dimension *h*/2 become first order cells; each of these first-order cells is divided into eight first-order elements with dimension *h*/4. The process is repeated to higher orders; B) variation of the logarithm of cumulative number of cubes with volumes *V* larger than comparative volume *v* ($\log[N(V > v)]$) against $\log(v)$ according to Eq. [3] (Methods section). In the graph, the values of *m* and D_f are also reported.

The model is based on the hypothesis that direct contact between two fragments of near equal size during the fragmentation process will result in the breakup of one of the blocks, then any initial particle distribution will evolve towards a distribution characterized by a minimum number of equal-sized particles at any scale. Furthermore, this model is based on the idea that it is unlikely that small fragments will break large fragments or that large fragments will break small ones[11,12]. According to the model shown in Fig. S3A, fragmentation starts from a cubic shape of size *h* and fragments into eight smaller cubes of size *h*/2. These smaller cubes are further fragmented following an iterative procedure to give cubes with size *h*/4, and so forth.

Considering the cumulative statistics of fragments resulting from the application of the above described iterative procedure (Fig. S3A), the cumulative number of fragments larger than a specified size for the three highest orders are N_{1c}= 2 for r= h/2, N_{2c}=14 for r=h/4, and N_{3c}=86 for r=h/8; N_{nc} is the cumulative number of the fragments equal to or larger than r_n, as required by Eq. [3] (Methods section). The cumulative statistics for the model illustrated in Fig. S3A, are given in Fig. S3B. Applying Eq. [4] (Methods section), the fractal dimension of fragmentation for this model gives a value of D_f=2.601. Such a value of D_f has been measured for a variety of rock types and appears to be a typical value for fragmentation of materials with solid-like state rheological behavior[11].

3D visualization of samples

3D movies of the microCT reconstructions of natural samples studied in this work are provided in order to better evaluate the distribution of trachybasaltic fragments in the

trachytic pumice. In particular, each of the three folders (Sample_073_UK, Sample_074_UK, and Sample_075_UK) contains three movies:

1) a full rotation (360°) movie showing the 3D reconstructed sample with the trachytic pumice and trachybasaltic fragments rendered in the grey and red colour, respectively;

2) a full rotation movie (360°) showing the segmented 3D volume in which only the distribution of trachybasaltic fragments are shown in the red colour;

3) a full rotation (360°) stereographic movie (red-cyan anagliphs) showing the distribution of trachybasaltic fragments. This movie needs be watched using red-cyan glasses in order to appreciate the 3D depth of the reconstructed volume.

References

1. Queiroz, G., 1997. Vulcão das Sete Cidades (S. Miguel, Açores): história eruptiva e avaliação do hazard. *Tese Doutoramento, DGUA, Ponta Delgada*, 226 pp.
2. Kueppers, U., Pimentel, A., Pacheco, J., 2009. The 16 ka eruption of Sete Cidades volcano, São Miguel Island (Azores, Portugal): Hazard assessment from mapping and simulation of tephra fall. In: *Union E. G. (ed.) EGU 2009. Vienna: Geophys. Res*. Abstr., p. 10810.
3. Queiroz, G., Gaspar, J. L., Guest, J.E., Gomes, A., Almeida, M.L., 2015. Eruptive history and evolution of Sete Cidades Volcano, São Miguel Island, Azores, in Gaspar, J. L., Guest, J.E., Duncan, A.M., Barriga, F.J.A.S., Chester, D.K. (eds). Volcanic Geology of São Miguel Island (Azores Archipelago). *Geol. Soc. London Mem.*, **44,** 87-104.

4. Moore, R.B., 1990. Volcanic geology and eruption frequency, São Miguel, Azores, *Bull. Volcanol.*, **52,** 602-614.
5. Moore, R.B., 1991. Geology of three late Quaternary stratovolcanoes on São Miguel, Azores, *US Geological Survey Bulletin, US Geological Service*.
6. Beier, C., Haase, K.M., Hansteen, T.H., 2006. Magma Evolution of the Sete Cidades Volcano, São Miguel, Azores. *J. Petrol.*, **47,** 1375-1411.
7. Queiroz, G., Pacheco, J.M., Gaspar, J.L., Aspinall, W.P., Guest, J.E., Ferreira, T., 2008. The last 5000 years of activity at Sete Cidades volcano (São Miguel Island, Azores): Implications for hazard assessment. *J. Volcanol. Geotherm. Res.*, **178,** 562–573. doi: 10.1016/j.jvolgeores.2008.03.001
8. Pacheco, J.M., Queiroz, G., Gonçalves, P., Gaspar, J.L.G., Ferrreira, T., 2005. Sete Cidades Volcano (S. Miguel, Azores): constraints to eruptive scenarios. In: *Union, E. G. (ed.) EGU 2005. Vienna: Geophys. Res*. Abstr., p. 09772
9. Matsushita, M.L., 1985. Fractal viewpoint of fracture and accretion. J. *Phys. Soc. Japan*, **54,** 857-860.
10. Turcotte, D.L., 1986. Fractals and fragmentation. *J. Geophys. Res.*, **91,** 1921-1926.
11. Turcotte, D.L., 1992. *Fractals and Chaos in Geology and Geophysics*. Cambridge University Press, Cambridge.
12. Sammis, C., King, G., Biegel, R., 1987. The kinematics of gouge deformation. *Pure Appl. Geophys*. **125,** 777-812.
